# Effect of Increased Intra-abdominal Pressure on the Esophagogastric Junction: A Systematic Review

**DOI:** 10.1097/MCG.0000000000001756

**Published:** 2022-09-07

**Authors:** Stefano Siboni, Luigi Bonavina, Benjamin D. Rogers, Ciara Egan, Edoardo Savarino, C. Prakash Gyawali, Tom R. DeMeester

**Affiliations:** * Division of General and Foregut Surgery, Department of Biomedical Sciences for Health, University of Milan, IRCCS Policlinico San Donato, San Donato Milanese; ‡ Humanitas University, Humanitas Research Hospital, Rozzano, Milan; § Gastroenterology Unit, Department of Surgery, Oncology, and Gastroenterology, University Hospital of Padova, Padova, Italy; † Division of Gastroenterology, St. Louis, MO; ∥ Emeritus, Keck School of Medicine, University of Southern California, Montague, MI

**Keywords:** intra-abdominal pressure, intragastric pressure, lower esophageal sphincter, crural diaphragm, esophageal manometry, high-resolution manometry, esophageal acid exposure, straight leg raise, gastroesophageal reflux disease

## Abstract

With the advent of high-resolution esophageal manometry, it is recognized that the antireflux barrier receives a contribution from both the lower esophageal sphincter (intrinsic sphincter) and the muscle of the crural diaphragm (extrinsic sphincter). Further, an increased intra-abdominal pressure is a major force responsible for an adaptive response of a competent sphincter or the disruption of the esophagogastric junction resulting in gastroesophageal reflux, especially in the presence of a hiatal hernia. This review describes how the pressure dynamics in the lower esophageal sphincter were discovered and measured over time and how this has influenced the development of antireflux surgery.

The existence of the lower esophageal sphincter (LES) was proven by Code who, in 1956, recorded a persistent high-pressure zone in the distal esophagus which relaxed with swallowing.^1^ The finding became the subject of intense in vivo and in vitro investigation. The precise physiological role and clinical implications in patients with gastroesophageal reflux disease (GERD) are still openly debated. Based solely on the LES tone, conventional manometric readings of the LES pressure failed to reliably segregate individuals with physiological reflux from those with pathologic levels. High-resolution manometry (HRM) has opened new perspectives by recognizing the contribution of both the LES as an intrinsic sphincter and the crural diaphragm (CD) as an extrinsic sphincter (Fig. 1). About 20% of the western population experience reflux symptoms at least weekly.^2^ Consequently, the modern diagnosis and treatment of GERD has become a priority for both gastroenterologists and surgeons.

**FIGURE 1:**
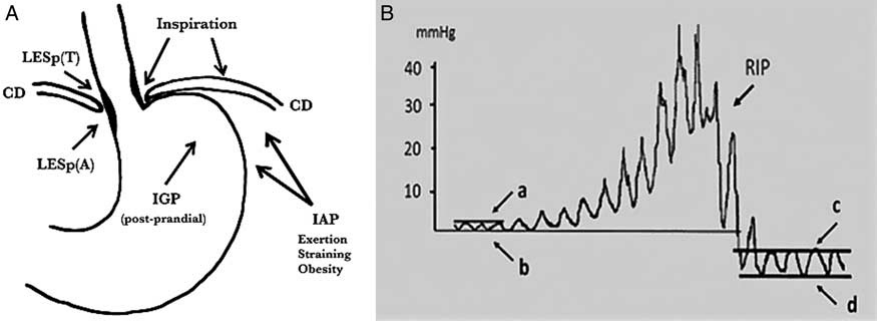
A, Effect of IAP and inspiration on the LES and CD in normal subjects. B, Normal LES pressure tracing indicating thoracoabdominal gradient fluctuations during respiratory phases. CD indicates crural diaphragm; IAP, intra-abdominal pressure; IGP, intragastric pressure; LESp, lower esophageal sphincter pressure; RIP, Respiratory Inversion Point.

It has been known that increased intra-abdominal pressure (IAP) can initiate GERD by equalizing gastric and esophageal pressure. This has stimulated studies by several investigators on the effects of straight leg raising (SLR), Valsalva maneuver, and the abdominal binders as maneuvers to increase IAP and challenge the antireflux barrier.

Aim of the current study was to assess the response of the LES to increased IAP. Our review showed that increased IAP is the major force responsible for an adaptive response of a competent LES. If the LES has been damaged, an increase in abdominal pressure can disrupt the gastroesophageal junction and result in gastroesophageal reflux. This is especially so in the presence of a hiatal hernia. Further, the review described how the pressure dynamics were discovered, measured, maintained, and identified when a surgical repair was needed.

Finally, this review looked at the past to gain a perspective on the present. It also represents a tribute to the extraordinary work done by pioneers and mentors of esophageal pathophysiology in light of the current knowledge obtained by HRM.

## METHODS

A systematic review was performed using the Cochrane, Embase, and PubMed databases according to the Preferred Reporting Items for Systematic Reviews and Meta-analyses (PRISMA) guidelines.^3^ Two authors (S.S. and C.E.) independently queried the databases with the following terms: “intra-abdominal pressure,” “esophageal manometry,” “provocative maneuvers,” “gastro-esophageal reflux disease,” “lower-esophageal sphincter,” “esophagogastric junction,” Abbreviations and synonyms were also included. Boolean operators AND, OR, and NOT were used. References from selected papers were analyzed to identify additional full-text papers. All original full-text English-written papers that included the relationship between IAP and LES were incorporated into the study. Papers not available in the full text were excluded. The following topics were analyzed and categorized in thematic sections: methodology used to increase IAP, LES response to IAP, relationship between LES and CD, effect of increased IAP on hiatal hernia, effect of increased IAP of esophageal acid exposure, use of IAP as a provocative maneuver, and the effect of increased IAP on the outcomes of antireflux surgery.

## RESULTS

**FIGURE 2:**
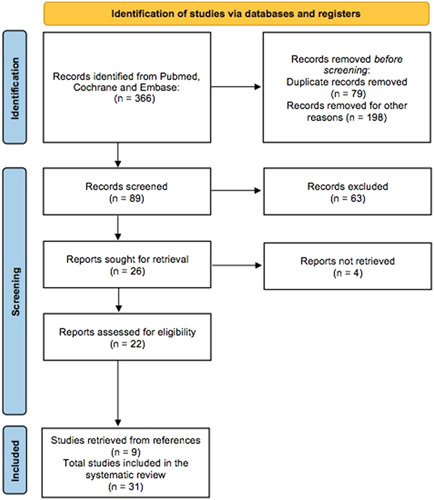
The Preferred Reporting Items for Systematic Reviews and Meta-analyses (PRISMA) flow chart.

Computerized search using selected criteria identified 366 studies. Of these, 198, consisting of abstracts, reviews, letters, and editorials, were excluded. Further, there were 79 duplicate publications and all were removed. The remaining 89 publications were screened and 22 met the inclusion criteria. The references of the eligible publications were then reviewed, and 9 additional manuscripts met the inclusion criteria. A total number of 30 studies were included in the final review (Fig. 2). According to the specific pertinence, each study was classified in 1 or more of the 7 thematic sections. The selected studies are chronologically summarized in Table 1.

**TABLE 1:** Timeline of Key Scientific Contributions and Pathophysiological Studies Investigating the Effect of IAP on the EGJ Barrier

Year	References	Main Findings
1956	Code et al^1^	First manometric evidence of the LES
1961	Nagler and Spiro^4^	Increased IAP through a pneumatic cuff. Two of 3 volunteers had increased intra-abdominal LES pressure
1964	Vanderstappen and Texter^5^	Differentiation from a crural (pinchcock) and a dome (IAP) action of the diagram
1965	Wankling et al^6^	LES pressure increase following IAP increase was seen in patients with normal LES pressure, regardless the presence of hiatal hernia. They hypothesized that increase IAP might be a provocative maneuver during esophageal manometry
1966	Lind et al^7^	Discovery of 2 high-pressure zones in patients with hiatal hernia. During abdominal compression, the region between LES and CD reached intragastric pressure. They concluded that LES rather than CD had the major role in competence
1968	Lind et al^8^	LES pressure did not differ in symptomatic, asymptomatic pregnant women, and controls
1968	Lind et al^9^	Decreased LES pressure during atropine infusion. Concluded that the intrinsic contraction of the LES was due to vagal reflex
1971	Cohen and Harris^10^	In 75 patients with and without hiatal hernia, the increased IAP generated an increased LES pressure in asymptomatic patients, regardless the presence of hiatal hernia
1972	Butterfield et al^11^	Introduction of the “common cavity test”. Increased esophageal pressure during IAP increase in symptomatic patients
1973	Alday and Goldsmith^12^	Degree of fundoplication has a direct relationship with restoration of a pressure gradient across the EGJ
1974	DeMeester et al^13^	Nissen fundoplication provides a greater increase of LES pressure and intra-abdominal LES length than Hill and Belsey operations. Abdominal compression by hand showed that the decrease of gastric pressure transmitted to the esophagus was greatest with the Nissen operation
1975	Dodds et al^14^	Comparison between 20 volunteers and 35 esophagitis patients. LES response to increased IAP was not affected by atropine infusion. Support of the main contribution of extrinsic factors to EGJ response to increased IAP
1979	DeMeester et al^15^	Clinical and in vitro study. Intra-abdominal LES length played a major role in sphincter competency
1979	Muller et al^16^	During abdominal loading, the tonic activity of diaphragm increased proportionally to the amount of the load
1980	Wernly et al^17^	Study combining IAP and 24-h pH monitoring. IAP increase became significant only in patients with severe LES alterations
1981	DeMeester et al^18^	Importance of phrenoesophageal ligament insertion to provide competency during IAP increase
1982	Joelsson et al^19^	Abnormal esophageal acid exposure on 24-h pH monitoring correlated to either an anatomic or functional LES dysfunction or a defective pump action of esophageal body
1986	Bonavina et al^20^	Mixed clinical and in vitro study. Higher prevalence of abnormal 24-h pH test in patients with defective sphincter
1989	Mittal et al^21^	Introduction of the 2-sphincter hypothesis: LES and CD are distinct sphincters that operate in synergy
1990	Mittal et al^22^	Electromyography and atropine infusion in 15 healthy subjects showed diaphragmatic activation during IAP increase
1993	Klein et al^23^	Demonstration of a high-pressure zone at the thoracoabdominal junction after esophagectomy reflecting the pinchcock effect of the CD
2011	Kwiatek et al^24^	3-dimensional HRM study on CD contribution to competence of the EGJ
2013	Louie et al^25^	Hiatal closure contributes more to restore LES pressure
2015	Lee and McColl^26^	Obesity and waist belt contribute to reflux through disruption of EGJ and IAP increase
2017	Mitchell et al^27^	Impaired clearance might be induced or worsened by increased high IAP, especially after meals
2020	Rogers et al^28^	First use of straight leg raise maneuver with HRM, significant association between increased esophageal pressure during leg raise and AET
2020	Stefanova et al^29^	Intraoperative EndoFLIP study in 100 patients who underwent Nissen, Toupet, or magnetic sphincter augmentation. Diaphragmatic repair and LES intra-abdominal relocation have greater effect on competency than sphincter augmentation
2021	Siboni et al^30^	Hiatoplasty contribution to EGJ barrier function after magnetic sphincter augmentation
2021	Gysen et al^31^	Introduction of gastrosphincteric pressure gradient to differentiate rumination from GERD patients
2021	Attaar et al^32^	Intraoperative EndoFLIP study in 97 patients. Hiatal repair provided a significant decrease in LES distensibility

AET indicates acid exposure time; CD, crural diaphragm; EGJ, esophagogastric junction; EndoFLIP, endoluminal functional lumen imaging probe; GERD, gastroesophageal reflux disease; HRM, high-resolution manometry; IAP, intra-abdominal pressure; LES, lower esophageal sphincter.

### Methodology Used to Increase IAP

Multiple methodologies have been evaluated for their ability to physiologically or artificially increase IAP. In the early stage of esophageal manometry, pneumatic cuffs and abdominal binders were studied extensively for their ability to provide a controlled external pressure on the abdomen. In 1961, Nagler and Spiro^4^ described 3 asymptomatic volunteers who underwent esophageal manometry during abdominal compression with a pneumatic cuff. In 2 of the 3 subjects, an increased pressure in the intra-abdominal segment of the LES was noted. A few years later, Cohen and Harris^10^ studied 75 patients, with or without hiatal hernia, during increased IAP from a Valsalva maneuver, an abdominal binder, or leg raising. No significant LES pressure differences were noted between the 3 modalities. Dodds et al^14^ first evaluated the effect different modalities had on IAP in a study of 20 normal volunteers and 35 patients with esophagitis. Abdominal compression was achieved using a pneumatic pressure cuff inflated to 50 and 100 mm Hg, while Valsalva and SLR maneuvers were both sustained for at least 20 seconds. Interestingly, while the gastric pressure increment was similar between the 3 methods, the LES-gastric pressure gradient was significantly higher during the SLR maneuver. The authors hypothesized that “the mechanical intrahiatal compression of the LES could create LES pressure changes that exceed those in gastric pressure.” From this point forward, SLR has been used as an alternative to abdominal binders to increase IAP. Thus, even though available data are scarce to draw robust conclusions, SLR may be an effective modality to study the LES response to increased IAP.

### LES Response to IAP

#### Pressure Response

Some studies have artificially increased IAP as a method to assess the physiological response of the LES in an attempt to define competence and predict esophageal pathology. In 1966, Lind et al^7^ used an abdominal binder in a cohort of patients without hiatal hernia and noted a pressure increase in the high-pressure zone corresponding to the CD-LES complex. They hypothesized that this response might be due either to the compression of the abdominal portion of the esophagus by the CD or by a contraction of the sphincter itself. To solve the dilemma, they added a subgroup of patients with hiatal hernia. In resting conditions, 2 high-pressure zones corresponding to LES and CD were recorded; conversely, during abdominal compression, the region between the high-pressure zones reached the intragastric pressure. The authors concluded that the LES, rather than the CD, plays a major role in maintaining the gastroesophageal pressure gradient, supporting the hypothesis of an intrinsic LES contraction.

**FIGURE 3:**
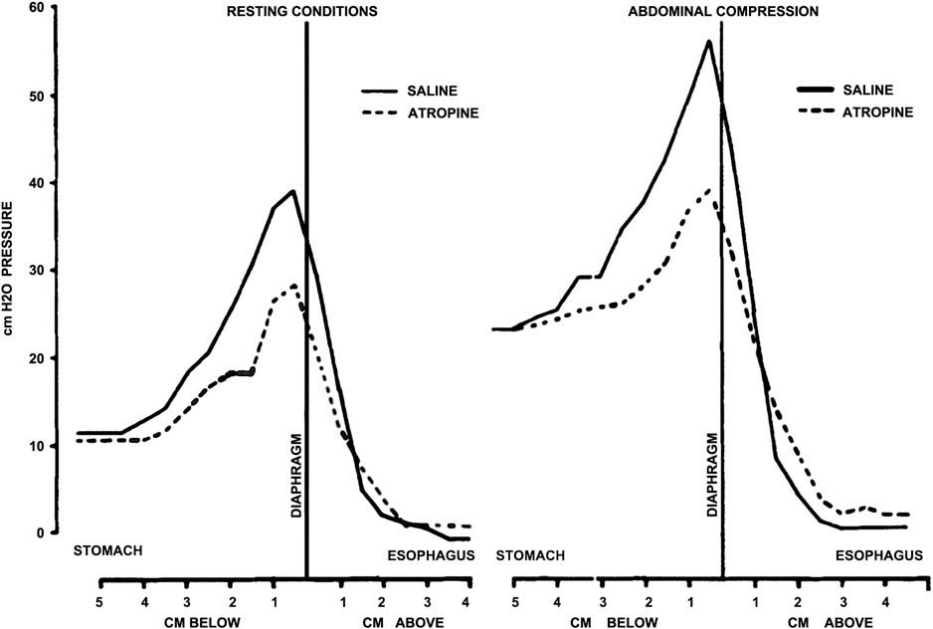
Decrease of lower esophageal sphincter pressure with atropine infusion during resting conditions and abdominal compression.^9^

An additional study demonstrated that LES pressure differed among symptomatic and asymptomatic pregnant women, regardless of the presence of a hiatal hernia.^8^ Further, an additional study was performed with the administration of atropine sulfate (0.025 mg/kg) in healthy subjects. During resting conditions, there was a pressure drop of 11.9 cm of water between the stomach and esophagus, but this difference increased to 20.1 cm of water during abdominal compression (Fig. 3). An interesting observation was that esophageal pressure increased during abdominal compression in some subjects. Based on these findings, the authors speculated that the intrinsic contraction of the smooth muscle in response to abdominal compression was related to a vagal reflex.^9^

**FIGURE 4:**
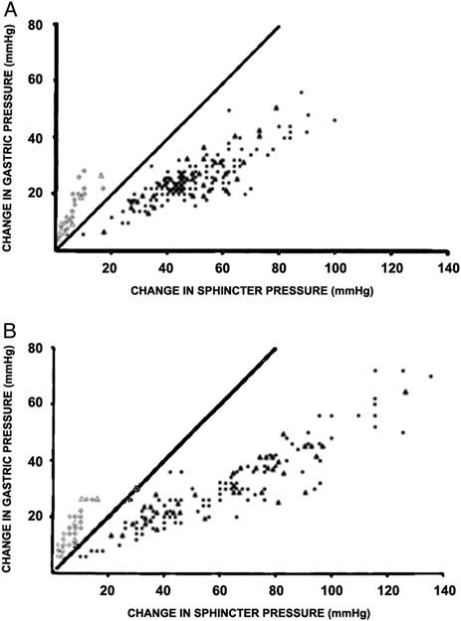
Relationship between increased intragastric and lower esophageal sphincter pressures in patients with gastroesophageal reflux disease symptoms (A) compared with asymptomatic controls (B).^10^

A study by Cohen and Harris,^10^ in 1971, further demonstrated that LES response to increased IAP is independent from the presence of hiatal hernia in asymptomatic subjects. There were significant differences in LES pressure change in patients with GERD symptoms compared with asymptomatic patients (Fig. 4), suggesting that LES pressure dynamics in response to abdominal strain might contribute to GERD. However, a few years later Dodds et al^14^ refuted this hypothesis showing that percent change and pressure profile of the LES response to SLR after atropine infusion was similar to baseline response before atropine infusion. Instead, they demonstrated that LES pressure increased significantly only during leg raise, rather than by abdominal compression or the Valsalva maneuver, indicating a mechanical barrier response rather than a smooth muscle response.

**FIGURE 5:**
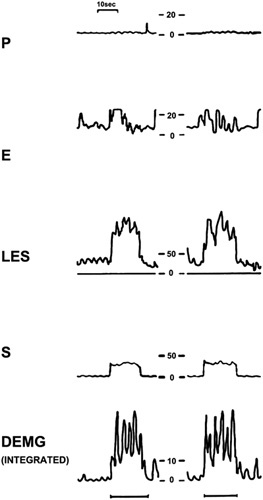
Modifications in esophageal (E) pressure, lower esophageal sphincter (LES) pressure, gastric (S) pressure, and diaphragm electromyogram (DEMG) during straight leg raise maneuver.^22^

In 1990, Mittal et al^22^ recorded diaphragm activation through electromyography (EMG) and LES pressure response via esophageal manometry during atropine infusion, SLR, abdominal binder application, and the Muller maneuver on 15 healthy subjects. The results showed a slow (2 to 5 s) but significant increase of the LES pressure (from 25 to 85 mm Hg) at the onset of the SLR maneuver, with a rapid fall at the end. Further, EMG demonstrated diaphragmatic activation, which disappeared at the end of the maneuver. During atropine infusion, resting LES pressure was diminished, whereas peak LES pressure during SLR and Muller maneuver were not different from the premedication period. Combining these findings, the authors concluded that the increase of LES pressure is mostly due to active diaphragmatic contraction during leg raise (Fig. 5).

In summary, these studies demonstrate that during increased IAP, LES, and/or CD tone increases and contributes to antireflux barrier competency. The differences between the studies may be related to patient selection or differing experimental protocols and measurement techniques.

#### Length Response

**FIGURE 6:**
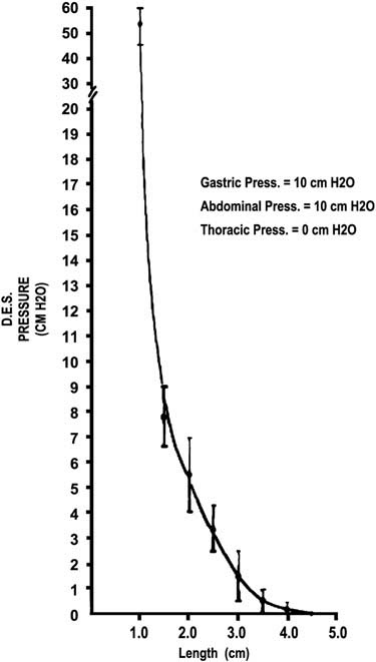
Relationship between lower esophageal sphincter pressure and length in providing lower esophageal sphincter competence.^15^ DES indicates Distal Esophageal Pressure.

DeMeester and colleagues^15^,^17^,^20^,^33^ proposed that the intra-abdominal LES length played a major role in preventing IAP from being transmitted into the esophagus. In an in vitro study,^15^ they showed that competency of LES as measured by amplitude of the distal high-pressure zone, without intrinsic tone, was related to its length. Under similar conditions, a longer abdominal esophageal length was necessary to maintain competence when IAP was increased. When artificially generated intrinsic tone was applied to the LES, the interrelationship between these 2 factors further clarified that the intra-abdominal esophagus is paramount to maintain competence. In fact, with LES length <1 cm, the intrinsic tone of the sphincter necessary to prevent reflux became infinite (Fig. 6). These findings emphasized the importance of adequate intra-abdominal esophageal length to compensate for increases in IAP, and led to the conclusion that a sufficient amount of intra-abdominal esophagus must be restored during antireflux surgery for benefit to be obtained. Ten years later, a review on this topic summarized the available evidence and reached similar conclusions.^33^ Since IAP compresses both the stomach and the intra-abdominal portion of the LES, an increase in IAP mechanically helps the sphincter to maintain competence. However, if the sphincter is entirely intrathoracic as in the presence of a hiatus hernia, this benefit is lost and the sphincter is unable to sustain increased IAP, thus increasing the likelihood of gastroesophageal reflux.

### Relationship Between LES and CD: the “Two-sphincter” Theory

One of the most controversial issues is whether the effect of increased IAP on the high-pressure zone is imputable to the intrinsic LES tone or is an extrinsic (ie, diaphragmatic) contribution. In 1964, Vanderstappen and Texter^5^ differentiated for the first time the crural (pinchcock) from the dome (IAP) action of the diaphragm. Intra-abdominal pressure was increased by both external abdominal compression and deep inspiration, resulting in a mean increase of fundic pressure of 12 and 15 mm Hg, respectively. During deep inspiration, the gastroesophageal pressure gradient was 32 mm Hg, while during external compression this gradient reached 20 mm Hg. The authors postulated 2 potential mechanisms for these findings, a rise in muscular tone of the LES itself or external compression by the CD. Since a pressure rise within the intra-abdominal LES segment caused by deep inspiration alone was unlikely, they concluded that augmentation of the high-pressure zone during deep inspiration might be caused by the peripheral (pinchcock) action of the diaphragm.

Two years later, Lind et al^7^ evaluated 24 normal volunteers and 9 hiatal hernia patients and showed that, during abdominal compression, pressure between the 2 high-pressure zones (representing the LES and the CD) was comparable to intragastric pressure in the presence of a hiatus hernia, while esophageal pressure proximal to the LES remained stable. The authors concluded that the gastroesophageal pressure gradient was maintained by the intrinsic tone of the LES, which was considered responsible for competency.

Muller et al^16^ studied the muscular tone of the diaphragm using EMG during abdominal compression and added important insights into the function of the diaphragm. Although the study cohort had only 3 subjects, the tonic activity of the diaphragm was increased during abdominal pressure loading, and was proportional to the amount of the load. Therefore, the authors hypothesized a “stretch reflex” of diaphragmatic muscle spindles contributing to diaphragmatic tone, even if they could not exclude other mechanisms such as a vagal reflex.

In 1990, Mittal et al^22^ added further knowledge to the role of the diaphragm by studying 15 healthy subjects with a manometric catheter equipped with 2 platinum electrodes to detect diaphragmatic EMG activity. During the SLR maneuver, gastric pressure reached a mean value of 30 mm Hg, while LES pressure increased up to 85 mm Hg. EMG showed an activation of the diaphragm during the maneuver, suggesting a significant contribution of the diaphragm to the pressure increase.

Overall, these studies emphasize the importance of the diaphragm in the barrier function of the esophagogastric junction (EGJ) during increased IAP. However, the lack of overlap of CD and LES in patients without hiatal hernia makes it difficult to assess, even with modern technology, which element contributes more to the barrier. Recent studies with 3-dimensional HRM have hypothesized that in patients with superimposed Lower Esophageal Sphincter and CD, the latter contributes with an asymmetric and vigorous pressure.^24^

### Effect of Increased IAP in Patients With Hiatal Hernia

The first study defining the effect of increased IAP in patients with a hiatus hernia was published by Wankling et al.^6^ Twenty asymptomatic controls and 24 patients with hiatal hernia were studied using abdominal compression via a pneumatic cuff (50 mm Hg). The study population was stratified by presence of hiatal hernia and resting LES pressure, thus dividing the population into normal or feeble sphincter. In the feeble sphincter group, abdominal compression increased esophageal pressure by 20 cm H_2_O, with a 5 cm decrease of pressure gradient between stomach and esophagus. These differences were not demonstrated in patients with normal sphincter pressure, regardless of the presence of hiatal hernia. The authors concluded that the presence of a hiatus hernia was not essential for reflux to occur, but the ability of the sphincter to maintain competence even when displaced into the thorax determined whether reflux occurred or not.

In 1971, Cohen and Harris,^10^ stratified 75 patients by GERD symptoms and hiatal hernia. Again, increase in gastric pressure exceeded the increase in LES pressure in patients with symptoms but not in asymptomatic patients with hiatal hernia, where the increase of LES pressure exceeded the increase in gastric pressure during Valsalva maneuver, SLR, or abdominal compression (Fig. 4). The authors concluded that a sliding hiatus hernia does not predispose to GERD, pressure surrounding the LES does not affect competence, and increase in LES strength secondary to increase in gastric pressure is not influenced by location of the LES in the abdomen or the chest.

**FIGURE 7:**
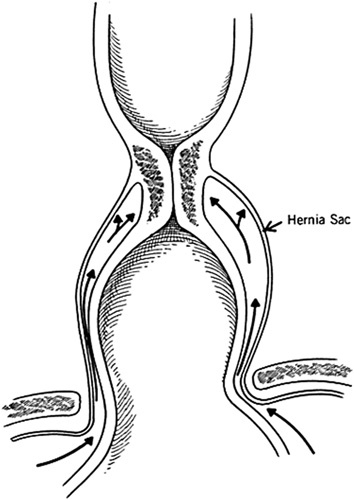
Diagram showing the effect of hiatal hernia sac on lower esophageal sphincter.^17^

In 1980, Wernly et al^17^ attempted to explain these findings using simultaneous esophageal manometry, 24-hour pH monitoring, and increased IAP. They calculated that there was a high number of increased IAP episodes through the study day, but only 8% of them induced a reflux episode. The key point of their discussion was that the distal portion of the esophagus in the hernia sac was exposed to abdominal pressure even in the presence of a hiatal hernia (Fig. 7). Based on the observation that >90% of episodes of increased IAP did not generate reflux even in patients with reflux, the authors concluded that only a severe mechanical or functional alteration of the cardia (ie, pressure of the sphincter <5 mm Hg and abdominal length <1 cm) resulted in reflux with increased IAP.

An explanation for the equivocal results regarding the role of the hiatus hernia was proposed by DeMeester et al.^18^ This study demonstrated the importance of the phrenoesophageal ligament, where high insertion of this ligament resulted in an adequate esophageal abdominal length likely because IAP is transmitted to the sphincter through the hernia sac (Fig. 7). Furthermore, DeMeester^33^ showed in an in vitro model that stepwise increase of IAP caused a stepwise increase in sphincter pressure only if the sphincter was placed in the abdominal cavity. In conclusion, isolated increase of IAP cannot predict reflux, even in the presence of a hiatus hernia, and patients with intact LES tone may be able to sustain IAP challenges even when the sphincter is intrathoracic.

### Effect of Increased IAP on Esophageal Acid Exposure

The first study to report a relationship between increased IAP and esophageal acid exposure was published by Vanderstappen and Texter^5^ in 1964. A simultaneous recording of intraluminal pressure and pH showed that acidification of the esophagus often occurred with an increased IAP. In normal subjects, reflux occurred only at the time of sphincter relaxation during deglutition. These findings emphasized the importance of the high-pressure zone and its capacity to adapt to increased IAP. Despite significant limitations (ie, low number of patients, rudimental technology, and absence of 24-h pH data), this study helped to define the concept of LES and its correlation with GERD.

Subsequently, Wernly et al^17^ focused on the relationship between IAP and 24-hour esophageal pH. IAP was monitored with a guard-ring tocodynamometer and pH with a standard pH probe. Only 8% of the IAP challenges induced a reflux episode. This rate, however, increased to 13% when only patients with defective LES were taken into account. The number of reflux episodes recorded during the study period was 2.7 per hour and only 38.7% of them were caused by an increased IAP. The authors concluded that the role of IAP was important in the genesis of reflux only if the LES was defective (pressure <5 mm Hg and intra-abdominal length <1 cm). Further, in an in vitro and clinical study, Bonavina et al^20^ showed a higher prevalence of abnormal 24-hour pH test in patients with intra-abdominal LES length <1 cm and LES pressure <6 mm Hg. However, the fact that patients with normal parameters still might have reflux indicated that there were other factors responsible for competence of the cardia, such as the degree of gastric dilatation. The authors concluded that individuals with either low LES pressure or short intra-abdominal LES length are unable to cope with the physiological increase of IAP caused by normal activities such as straining or a change in body position, can result in gastroesophageal reflux.

In 1982, Joelsson et al^19^ explored a larger population of GERD patients to characterize the pathogenesis of reflux. They found that increased esophageal acid exposure resulted from multiple mechanisms, including anatomic or functional LES failure and a defective pump action of the esophageal body. More recently, Mitchell et al^27^ showed that impaired esophageal clearance might be induced or worsened by increased IAP, especially after meals. Further, the application of a waist belt increased the rate of transient LES relaxations associated with reflux and impaired the esophageal clearance of refluxed acid. Finally, Lee and McColl^26^ speculated that in patients with either central or waist belt obesity, an intrasphinteric reflux might also occur which may be associated with the current rise of adenocarcinoma of the EGJ. These studies demonstrate that increased IAP may be an initiator of GERD. Therefore, challenging the EGJ with a provocative maneuver during HRM could facilitate the identification of true GERD patients.

### Rising IAP to Improve the Diagnostic Yield of Esophageal Manometry

The first study to validate the SLR maneuver as an adjunctive “stress” test to prove the competency of the LES was published by Wankling et al^6^ in 1965. An increase in esophageal pressure was seen only when LES pressure did not increase in response to the increased IAP, both in volunteers and hiatal hernia patients. Although this study involved only a few subjects, there was a significant correlation with intraesophageal pressure increase in patients with a feeble sphincter. On the contrary, no correlation was found in patients with or without hiatal hernia. The authors concluded that the addition of abdominal compression allowed better assessment of the functional integrity of LES.

In 1972, Butterfield et al^11^ introduced the concept of a “common cavity” test, where gradual abdominal compression resulted in a higher increase in esophageal pressure in symptomatic patients compared with asymptomatic patients (34 vs. 9.9 mm Hg, *P*<0.001). Interestingly, resting LES pressure was similar in the 2 groups but increased significantly in asymptomatic compared with symptomatic patients during abdominal compression. Since esophageal pressure equalized gastric pressure during abdominal compression, they called this the “common cavity” phenomenon, and proposed gradual abdominal compression as a provocative test during esophageal manometry. However, the common cavity test was not widely adopted in clinical practice, and only few studies have reported its utility to validate the effectiveness of antireflux surgery.^34^,^35^

In 1975, Dodds et al^14^ demonstrated the “common cavity” phenomenon in 6/35 patients with esophagitis during a 100 mm Hg abdominal compression. Surprisingly, they did not give much significance to this finding, stating that “abdominal compression did not separate most patients with esophagitis from asymptomatic volunteers.” They also suggested that abdominal compression provided further diagnostic information only when a “common cavity” phenomenon was present, thus minimizing the importance of the SLR as a possible provocative test during esophageal manometry. Conversely, in all other studies, a positive “common cavity” test was associated with symptoms. Given the lack of precise cutoffs and the relative low sample size, Butterfield and colleagues’ study did not gain popularity, and the scientific community lost a promising provocative maneuver that could have improved the accuracy of esophageal manometry.

### Increased IAP and Outcomes of Antireflux Surgery

There has been a mix of serendipity and pure science in the evolution of modern antireflux surgery. The surgical community watched closely and often contributed to the understanding of esophageal pathophysiology with the aim to improve surgical outcomes. Since the first description of hiatal hernia repair published in 1919 by Soresi,^36^ diaphragmatic repair has represented the mainstay of treatment to keep the stomach within the abdominal cavity. However, it was only in 1951 that Allison^37^ and Barrett^38^ found a physiological correlation between hiatal hernia and GERD, giving birth to the true antireflux surgery era. However, there were important conceptual differences between the 2 pioneers’ views. Allison believed in the pinchcock function of the CD and therefore focused his operation on hiatal repair and fixation of the phrenoesophageal ligament to the diaphragm. In contrast, Barrett thought that the most effective physiological antireflux mechanism was the angle of His, therefore its restoration became the target of antireflux surgery. Later, Skinner and Belsey^39^ and Hill^40^ based their operation on this mechanistic hypothesis. In the meantime, Rudolf Nissen developed his technique of 360-degree fundoplication by focusing on restoration of the intra-abdominal esophageal segment and augmentation of LES pressure. Interestingly, Nissen developed his technique serendipitously, by realizing that use of the residual gastric fundus to protect the esophagogastric anastomosis from leaks prevented postoperative symptomatic GERD.

In 1973, Alday and Goldsmith^12^ demonstrated in experimental model a direct relation between the degree of fundoplication and its efficacy in maintaining competence and a low esophageal pressure during progressive gastric compression. At a minimum, a 270-degree wrap was necessary to maintain an effective pressure gradient across the EGJ. Furthermore, in a prospective study comparing Nissen, Hill, and Belsey operations, DeMeester et al^13^ demonstrated that Nissen fundoplication was superior in increasing LES pressure and placing the sphincter in the positive abdominal environment. The abdominal compression test performed by hand clearly demonstrated that the amount of gastric pressure transmitted to the esophagus was greatest with the 360-degree fundoplication. Therefore, the Nissen operation rapidly became the gold standard in the surgical treatment of GERD and hiatal hernia. Further evolution of the technique led to shortening the fundoplication to 2 cm. in length and calibrating its circumference with a 60 French bougie to prevent dysphagia.^41^

In 1989, Mittal et al^21^ developed the “two-sphincter” hypothesis and revisited the contribution of CD to the competence of the EGJ. In 1993, Klein et al^25^ described a sphincter-like high-pressure zone at the thoracoabdominal junction in patients who underwent esophagectomy and gastric conduit replacement. This further supported the hypothesis that the pinchcock mechanism of the CD represents a distinct barrier to reflux in addition to the intrinsic LES.

Louie et al^23^ confirmed that the crural plasty contributes more to restore LES pressure than LES intra-abdominal length, while fundoplication is more active in avoiding LES shortening and spontaneous LES relaxation. More recently, studies assessing the separate effect of hiatal repair and fundoplication using the endoluminal functional lumen imaging probe (EndoFLIP) suggested that hiatal repair and relocation of the LES into the abdomen are more important than fundoplication to restore competency to the EGJ.^29^,^32^ All the above findings support the hypothesis that the LES and CD function as distinct sphincters and operate in synergy to prevent reflux; therefore, to be effective, antireflux surgery must restore competence of both sphincters.

## DISCUSSION

This review shows that LES response to increased IAP has been a topic of interest for several decades. Integration of experimental data into clinical practice has demonstrated that increasing IAP is a simple and effective method to assess the LES. The information obtained may prove useful to augment diagnostic accuracy in patients with suspected GERD and to clarify the indications for antireflux surgery.

A common finding throughout the studies was an increased LES pressure in response to increased IAP in asymptomatic volunteers and patients with normal LES pressure and length. In symptomatic GERD patients, the LES pressure increase was more unlikely and was independent of the presence of hiatal hernia.^6^,^10^ Assessment of the separate contributions of LES and CD to competence of the EGJ was challenging, given the anatomic proximity of the 2 sphincters.^5^,^7^ Studies with atropine infusion, EMG, and 3-dimensional esophageal HRM seem to suggest that the CD plays a crucial role in increasing the LES-CD complex pressure.^9^,^22^,^24^ This is supported by more recent evidence that hiatal repair is an essential component of antireflux surgery,^25^,^29^,^30^,^32^ confirming the “two-sphincter hypothesis” originally proposed by Mittal et al.^21^

Increased IAP is certainly not the only determinant of gastroesophageal reflux.^33^ Rather, increased IAP is part of a spectrum of pathophysiological mechanisms including transient LES relaxations, impairment of esophageal clearance, increased thoracoabdominal pressure gradient,^42^ and the gastric acid pocket.^43^ This could explain why the outcomes of current antireflux procedures, such as magnetic sphincter augmentation, are improved by routine mediastinal dissection and posterior crural repair.^30^,^44^,^45^,^46^ Disruption of the EGJ frequently occurs in patients with central obesity which is associated with increased IAP.^26^ This could also explain why Roux-en-Y gastric bypass is considered more effective than fundoplication in reducing GERD symptoms and recurrence rates in morbid obese patients.^47^

Last, HRM-impedance studies on patients with rumination syndrome have emphasized the role of the gastrosphincteric pressure gradient rather than the gastroesophageal pressure gradient as an independent factor of backward flow.^31^ This supports the bulk of evidence that increased IAP promotes reflux mainly in patients with disrupted EGJ.

Despite the important limitations due to heterogeneity of the experimental models and the fact that active or passive IAP increase might activate different physiological patterns,^14^ several studies demonstrate the utility of adding a stress test as a provocative maneuver during esophageal manometry. The reasons for this assumption are 2-fold. First, assessing LES response to increased IAP might help to better characterize sphincter function and its ability to endure a pressure challenge.^7^ Second, the increase in esophageal pressure (“common cavity” test) might help to identify patients with a defective LES.^11^ Furthermore, the addition of a Muller maneuver might help to quantify the single contribution of CD to competency.

**FIGURE 8:**
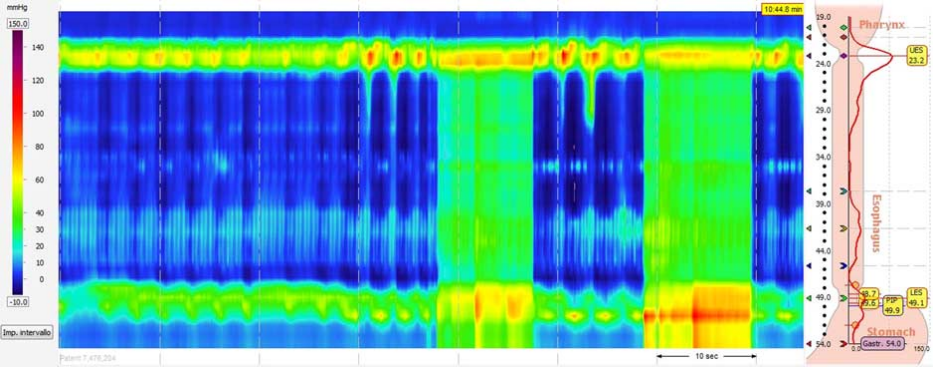
High-resolution manometry plot showing increased esophageal pressure during straight leg raising. LES indicates lower esophageal sphincter; UES, upper esophageal sphincter.

With the advent of HRM and the recognition that the CD is a crucial component of the EGJ barrier, there has been a better overall understanding of GERD pathophysiology. Rogers et al^28^ first demonstrated a significant association between SLR and esophageal acid exposure time. However, strong evidence in terms of thresholds and modalities to establish a reproducible pressure increase is still lacking. A multicenter study to determine the optimal cutoff of increased esophageal pressure during HRM with SLR is underway^48^ (Fig. 8).

## CONCLUSIONS

Increasing IAP during esophageal manometry is a useful and practical adjunctive provocative maneuver that might help to better characterize the GERD phenotype in clinical practice. Further translational research and standardization of HRM protocols is needed to improve the diagnostic yield and to improve patient selection for antireflux surgery.
